# *Boswellia serrata* Extract Containing 30% 3-Acetyl-11-Keto-Boswellic Acid Attenuates Inflammatory Mediators and Preserves Extracellular Matrix in Collagen-Induced Arthritis

**DOI:** 10.3389/fphys.2021.735247

**Published:** 2021-09-28

**Authors:** Muhammed Majeed, Kalyanam Nagabhushanam, Lincy Lawrence, Rameshprabu Nallathambi, Varadharajan Thiyagarajan, Lakshmi Mundkur

**Affiliations:** ^1^ Sami-Sabinsa Group Limited, Bangalore, India; ^2^ Sabinsa Corporation, East Windsor, NJ, United States

**Keywords:** inflammation, autoimmunity, matrix proteins, collagen-induced arthritis, acetyl-11-keto-β-boswellic acid, *Boswellia serrata*

## Abstract

*Boswellia serrata* extracts have been traditionally employed for the treatment of inflammatory diseases. In the present study, we have evaluated the mechanism of activity of Boswellin Super^®^ FJ (BSE), a standardized extract of *B. serrata* containing not less than 30% 3-acetyl-11-keto-β-boswellic acid along with other β-boswellic acids. The *in vitro* anti-inflammatory activities were carried out in RAW 264.7 macrophages or human peripheral blood mononuclear cells stimulated with bacterial lipopolysaccharides (LPS) and treated with 1.25-5μg/ml BSE. The anti-arthritic activity of the extract was evaluated in a rat model of collagen-induced arthritis. BSE at 40 and 80mg/kg and celecoxib 10mg/kg were orally dosed for 21days. BSE showed significant (*p*<0.05) inhibition of inflammation (TNF-α, IL-6, nitric oxide, and COX-2 secretion) and downregulates the mRNA levels of TNF-α, IL-6, IL1-β, and inducible nitric oxide synthase in macrophages. BSE treatment reduced the levels of phosphorylated-NF-κB (P65), suggesting an anti-inflammatory activity mediated by blocking this key signal transduction pathway. In addition, BSE showed inhibition (*p*<0.05) of collagenase, elastase, hyaluronidase enzymes, and a reduction in reactive oxygen species and matrix-degrading proteins in RAW 264.7 macrophages stimulated with LPS. BSE treatment significantly (*p*<0.05) reduced the arthritic index, paw volume, and joint inflammation comparable to celecoxib in collagen-induced arthritis (CIA) in rats. The circulating anti-collagen antibodies were reduced in BSE and celecoxib-treated animals as compared to the CIA. In confirmation with *in vitro* data, BSE showed a significant (*p*<0.05) dose-dependent effect on C-reactive protein, prostaglandin E2, and erythrocyte sedimentation rate, which is widely used as a blood marker of inflammation. Further, BSE treatment suppressed the cartilage oligomeric matrix protein and significantly enhanced the hyaluronan levels in synovial fluid. As observed by collagen staining in joints, the loss of matrix proteins was lower in BSE-treated animals, suggesting that BSE could preserve the extracellular matrix in RA. The extract showed inhibition of collagenase enzyme activity *in vitro*, further strengthening this hypothesis. BSE treatment was found to be safe, and rats displayed no abnormal behavior or activities. The results suggest that Boswellin Super^®^ mediates its activity by preserving matrix proteins, reducing pro-inflammatory mediators, and oxidative stress.

## Introduction

Rheumatoid arthritis (RA) is a chronic, inflammatory, and autoimmune disease, with a global prevalence estimated to range from 0.24–1% in different countries (Safiri et al., 2019). It is characterized by inflammation and swelling of the peripheral joints, pain, and articular cartilage damage. Synovial hyperplasia and tissue inflammation are the characteristic features of the disease. The synovium is also the source of various proteins and proteases that degrade articular cartilage and activate osteoclasts, leading to bone erosions, typically at the junction of bone and cartilage (Kyburz and Finckh, 2013).

During synovial hyperplasia, monocytes recruited into the synovium secrete cytokines and chemokines, inducing synovial fibroblast proliferation, pro-inflammatory cytokine production, matrix-degrading enzymes secretion, and sustained synovial hypertrophy (Bartok and Firestein, 2010). The cytokines, such as the receptor activator of nuclear factor-kappa-β ligand and granulocyte-macrophage colony-stimulating factor, control the osteoclast differentiation. Other inflammatory cytokines present in the synovium are tumor necrosis factor-alpha (TNF-α), interleukin (IL)-1β, and IL-6, contributing to bone erosion. Nitric oxide (NO), reactive oxygen species (ROS), prostaglandins, leukotrienes, platelet-activating factor, and enzymes [lipoxygenases, cyclooxygenases (COX-1 and COX-2), and phospholipases] are other inflammatory markers in RA. Abnormalities in the immune system leading to the presence of autoantibodies known as rheumatoid factors and immigration of T and B cells into the synovium are characteristic features of RA (Scherer et al., 2020).

RA is clinically managed using nonsteroidal anti-inflammatory drugs, corticosteroids, disease-modifying anti-rheumatic drugs (DMARDs), and biological response modifiers associated with several adverse effects (Schaffer et al., 2006). Conventional DMARDs, including methotrexate, hydroxychloroquine, and sulfasalazine, are widely used in RA therapy. Biologics are engineered proteins that target cytokines, or inflammatory cells or pathways related to tissue damage. The most common biologics are TNF-α antagonist like etanercept (Enbrel^®^) infliximab (Remicade^®^) adalimumab (Humira^®^), certolizumab pegol (Cimzia^®^), and golimumab (Simponi^®^). Other biologics include IL-1 inhibitor anakinra (Kineret^®^) and T-cell co-stimulation blocker abatacept (Orencia^®^). The patient response to DMARD is slow in many cases, and biologics are associated with adverse effects like susceptibility to infection and malignancies (Curtis and Singh, 2011).

Natural products and supplements could reduce the pain and morbidity associated with RA (Yang et al., 2016). The extracts of *B. serrata* (Family: Burseraceae) are widely used in traditional medicine due to their anti-oxidative, anti-inflammatory, and anti-arthritic properties (Ahmed et al., 2013; Mannino et al., 2016; Majeed et al., 2020a). Boswellic acids (BAs) are triterpenes present in the oleo gum resins of *Boswellia* species. Around 12 different pentacyclic triterpenes (BAs) have been identified (Buchele and Simmet, 2003). Among them, β-boswellic acid (BBA) and 3-acetyl-11-keto-beta-boswellic acid (AKBBA) have received significant pharmacological interest (Ammon, 2016). BAs have been reported to reduce the inflammation and associated markers in both RA and OA in clinical studies. AKBBA is the most potent inhibitor of 5 lipoxygenases (5-LO) and the leukotriene-mediated inflammatory pathways (Schweizer et al., 2000). Apart from leukotriene inhibition, AKBBA inhibits the COX-1 activity in human platelets, suppresses NF-κB activation and pro-inflammatory cytokines.

Although the *Boswellia* extracts with different BAs have been extensively studied, comprehensive evaluation of standardized extracts is scarce. Consistency of the chemical constituents of the extract is ensured by standardization. It is essential to study the activity of the standardized extract to confirm their safety and efficacy as they differ from the total plant extract which is used in traditional medicine. In the present study, we evaluated the mechanism of activity of Boswellin Super^®^ FJ (BSE), a standardized extract of *Boswellia serrata* containing not less than 30% 3-acetyl-11-keto-β-boswellic acid along with other β-boswellic acids, *in vivo* in a collagen-induced arthritis model. The safety of this composition has been established earlier (Majeed et al., 2020b).

## Materials and Methods

### Reagents

Freund’s complete adjuvant (AR001), collagen type II, and calcium ionophore A23187 (C7522) were procured from Sigma-Aldrich (St. Louis, MO, United States). Dulbecco’s modified minimal essential medium (DMEM) from Life Technologies (CA, United States) and fetal bovine serum (FBS) from GIBCO/Invitrogen (Carlsbad, CA, United States). DuoSet and Quantikine ELISA kits were from R&D Systems (Minneapolis, Minnesota, United States).

### Boswellia Extract

Boswellin^®^ Super (BSE) FJ is a standardized extract of *B. serrata*.

### Cell Culture

Mouse macrophage cell line (RAW 264.7) was purchased from National Centre for Cell Science (Pune, India) and maintained as a monolayer culture in DMEM, supplemented with 10% (v/v) FBS at 37°C in a humidified 5% CO_2_ incubator. Cytotoxicity/cell viability of both PBMC and RAW264.7 was measured after 24h of exposure to BSE by MTT assay (Liu et al., 2018).

### Isolation of PBMC

The human blood sample (5ml) was collected from one of the manuscript authors as a volunteer with informed consent in EDTA vacutainer tubes. The sample was diluted with an equal volume of RPMI media and overlayed on Histopaque. The tubes were centrifuged at 400 x g for 30min at room temperature. The peripheral blood mononuclear cells (PBMCs) were aspirated from the buffy coat in the Ficoll-plasma interface, washed with PBS, and resuspended in Hanks balanced salt solution. Cell viability and count were determined using the trypan blue dye exclusion method (Grievink et al., 2016).

### Antioxidant Assay

RAW 264.7 (5×l0^4^ cells/well) macrophage cells were seeded in 96 well black microplates and allowed to grow overnight. Cells were induced with H_2_O_2_ (25mm) and treated with different concentrations of BSE in DMEM 1% FBS for 4hours. Freshly prepared DCFH-DA reagent (10μg/ml) was added to all the wells. The fluorescence was documented at a wavelength of 485:520 (Ex: Em) nm, after 30min incubation at 37°C using the BMG FluoStar Optima microplate reader. The ROS scavenging percentage was calculated with respect to the fluorescence intensity of H_2_O_2_-treated control cells.

### Anti-inflammatory Assay

RAW 264.7 (8×l0^5^ cells/well) macrophage cells were seeded in six well plates and allowed to grow overnight. Cells were induced with LPS (500ng/ml) and or without different BSE concentrations for 24h. The cell supernatant was collected, and the cells were lysed using RIPA buffer (Hi-Media, India). Protein concentration in cell lysates was estimated by the Bradford method (Sigma, United States). The culture supernatants were used for nitrite estimation using the Griess reaction. The cell lysates were used to analyze the presence of IL1-β using a DuoSet ELISA kit, according to the manufacturers’ instructions (R&D Systems, Minneapolis, Minnesota, United States).

Alternately, human PBMC purified from healthy adults (5×10^5^ cells/well) were seeded in 96 well plates and stimulated with LPS (500ng/ml) in the presence of BSE for 24h. The supernatants were used for TNF-α and IL6 estimation using ELISA kits as per the manufacturer’s instruction (R&D Systems, Minneapolis, Minnesota, United States).

### Prostaglandin Estimation

Prostaglandin was estimated using a commercial kit (Quantikine ELISA kit, R&D Systems, Minneapolis, Minnesota, United States), using the 24h LPS and sample-treated PBMC cells (5×10^5^ cells/well) supernatant. The concentration of prostaglandin was calculated based on the standard provided in the kit.

### LTB4 Estimation

PBMC (5×10^5^ cells/well) were treated with sample along with Calimycin (10μm) and incubated for 30min at 37°C. The incubation was stopped by rapid cooling in an ice bath followed by centrifugation at 250g for 10min at 4°C. The concentrations of LTB4 in cell supernatants were measured by ELISA (R&D Systems, Minneapolis, Minnesota, United States) as per the manufacturer’s instructions. The cell supernatants were stored at −20°C until assay. The percentage inhibition of LTB4 was calculated to Calimycin-treated control (Kovács et al., 2014).

### Nitrite Determination

The nitrite accumulated in the culture medium was measured as an indicator of NO production based on the Griess reaction. Briefly, 50μl of cell culture medium was mixed with 50μl of Griess reagent I [1% (w/v) sulfanilamide in 5% (v/v) phosphoric acid] and 50μl Griess reagent II [0.1% (w/v) N-(1-Naphthyl) ethylenediamine dihydrochloride], incubated at room temperature for 10min, and then, the absorbance was taken at 540nm.

#### Collagenase Inhibition

Inhibition of collagenase enzyme was estimated by using the Enzchek^®^ gelatinase/collagenase assay kit, Invitrogen, United States. Type IV collagenase enzyme from *Clostridium histolyticum* with DQ^™^ gelatin was used as the substrate. Different concentrations of BSE (80μl) were preincubated with 20μl of gelatin substrate (12.5μg/ml). A volume of 100μl of the collagenase enzyme solution was added (final concentration of 0.4U/ml), and the fluorescence intensity was measured at 485nm and 520nm after 30min. Enzyme activity of control (buffer) was recorded as a negative control. The extent of inhibition was calculated by using the equation, [(B-BC) -(T-C)/(B-BC)×100], where B is the fluorescence in the presence of the enzyme, BC is the fluorescence in the absence of the enzyme, T is the fluorescence of enzyme activity in the presence of BSE, and TC is the fluorescence of the BSE alone.

#### Hyaluronidase Inhibition

The assay was performed following the method as described by Tung et al. (1994). A volume of 50μl hyaluronidase (20 Uml^−1^) in enzyme diluent (20mm sodium phosphate with 77mm sodium chloride and 0.01% (w/v) Bovine serum albumin, pH 7.0 at 37°C) was mixed with 50μl of different concentrations of BSE diluted with enzyme diluent followed by incubation at 37°C for 10min. The reaction was then initiated by the addition of 100μl of hyaluronic acid (0.03%) prepared in 300mM sodium phosphate buffer, pH 5.35 at 37°C as substrate solution and incubated at 37°C for 45min. Cetylpyridinium chloride (1ml) was used to precipitate the undigested hyaluronic acid. After incubation at room temperature for 10min, 200μl of each sample was transferred to 96 well microplates, and the absorbance of the reaction mixture was measured at 600nm using a microplate reader (Sunrise, TW, Tecan). All solutions were prepared freshly. The absorbance in the absence of enzyme was used as a control value for maximum inhibition. The inhibition percentage was calculated using the formula (Absorbance of Control-Absorbance of Sample/Absorbance of Control) × 100.

#### Elastase Inhibition

RAW 264.7 cells at a density of 6×10^5^ were seeded in a six well plate and incubated overnight and stimulated with 50ng/ml LPS along with different concentrations of BSE. The culture medium was collected after 16h to check the elastase enzyme activity in the supernatant. A volume of 50μl of assay buffer was preincubated with 50μl of elastin substrate (25μg/ml) in a 96 well black microtiter plate. 100μl of the culture supernatant was added fluorescence intensity was measured at 485nm and 520nm after 30min. The percentage inhibition of enzyme activity was calculated compared to the fluorescence intensity of LPS-treated control cells.

### MMP Activity (Zymography)

RAW 264.7 cells (6×10^5^) in a six well plate and incubated overnight for cell adhesion. The cells were stimulated with 50ng/ml LPS along with different concentrations of BSE. Culture medium or cell lysate was collected after 16h for Matrix metalloproteinase (MMP) gelatin zymography. Samples were mixed in Laemmli buffer lacking 2-mercaptoethanol and incubated at room temperature for 15min for gelatin zymography. Equal amounts of protein were loaded and separated on 8% SDS-PAGE containing 0.1% gelatin from bovine skin, type B (Sigma-Aldrich). Gels were washed for 20min, thrice in zymogram wash buffer (2.5% Triton X-100, 50mm Tris-HCl, pH 7.5, 5mm CaCl_2_, and 1μm ZnCl_2_) and incubated overnight at 37°C in zymogram developing buffer (2.5% Triton X-100, 50mm Tris-HCl, pH 7.5, 5mm CaCl_2_, 1μm ZnCl_2_, and 150mm NaCl). Gels were then stained with 0.5% Coomassie Brilliant Blue R-250 (Bio-Rad) for 1h and destained twice for 30min in 10% acetic acid and 40% methanol solution.

### Gene Expression by qRT-PCR

RAW 264.7 cells were treated with LPS as described in the earlier sections (6×10^5^ in a six well plate and stimulated with 50ng/ml LPS along with different concentrations of BSE). After 16h of treatment, total cellular RNA was isolated using Trizol reagent^®^ (Ambion, Life Technologies), followed by RNase-free DNase I treatment (Thermo Fisher Scientific) to remove any genomic DNA. One microgram of total RNA was reverse transcribed into cDNA using the revert-aid first strand cDNA synthesis kit (Thermo Fisher Scientific), and quantitative real-time PCR (qRT-PCR) was performed using the SYBR Green qPCR master mix (Thermo Scientific) using Light cycler 96 (Roche Life Science). β-actin gene expression was used as housekeeping control. The following primers were used for the analysis.


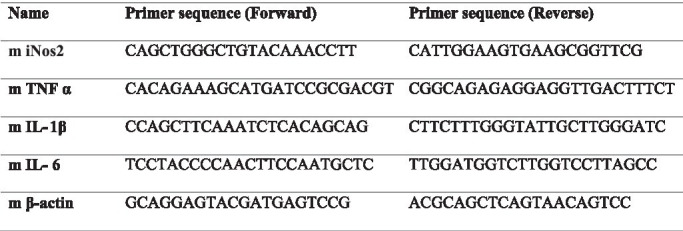


### Immunoblotting

Cellular protein (75μg) was loaded per lane in denatured 10% polyacrylamide gel (SDS-PAGE), transferred to a polyvinylidene difluoride membrane (Invitrolon^™^ PVDF, Thermo Fisher Scientific, United States). Membranes were incubated with anti-mouse primary antibodies against total and p-NFκB and β-actin (Cell Signaling Technology, Europe) for 18h at 4°C and horseradish peroxidase-conjugated secondary antibody (G-Biosciences, United States) for 2h at 37°C. ECL (Pierce ECL plus, Thermo Scientific, United States) was used to detect the immunoreactive proteins. The blots were quantified using ImageJ software (version 1.52a, National Institute of Health, United States).

### Animals and Experimental Design

Wistar rats were housed under standard air-conditioned laboratory conditions. The temperature was maintained at a maximum: 24°C and a minimum of 23°C and relative humidity at a maximum: 63% and a minimum of 48% with 12h light and 12h dark cycle. The maximum and minimum temperature and relative humidity in the experimental room were recorded once daily. The male Wistar rats (8weeks weighing 220–240g) were randomized into five groups of six animals, as shown in the experimental design. CIA was induced in Groups 2–5, and treatments were given by oral gavage. The experiment was carried out in accordance with the guidelines for animal experimentation of the Committee for the Purpose of Control and Supervision of Experiments on Animals (CPCSEA), India, with approval from the Institutional Animal Ethics Committee (approval no. 1165/PO/RcBiBt-S/NRc-L/08/CPCSEA).

### Collagen-Induced Arthritis

Chicken type II collagen (CII, 2mg/ml in 0.1M acetic acid, dissolved overnight at 4°C) was emulsified in an equal volume of complete Freund’s adjuvant (CFA) prepared by dissolving heat-inactivated Bacillus Calmette-Guerin in IFA to a final concentration of 0.5mg/ml. Rats were immunized with 0.2ml of CII emulsion at the base of the tail by the intradermal route. On day 7, a booster dose was administered (Rosloniec et al., 2001). Immunized rats received BSE (40 and 80mg/kg per day or celecoxib 10mg/kg by oral gavage) or vehicle, starting from day 0 to 21. The test formulations were suspended in 0.5% sodium carboxymethylcellulose and dosed at the volume of 10ml/kg body weight. At the end of the study, rats were euthanized by decapitation by cervical dislocation as per the American Veterinary Medical Association guidelines by a trained veterinary physician (Leary et al., 2020). Blood (1ml) and synovial fluid (0.8–1ml) were collected from all the animals for biochemical analysis.

### Experimental Design


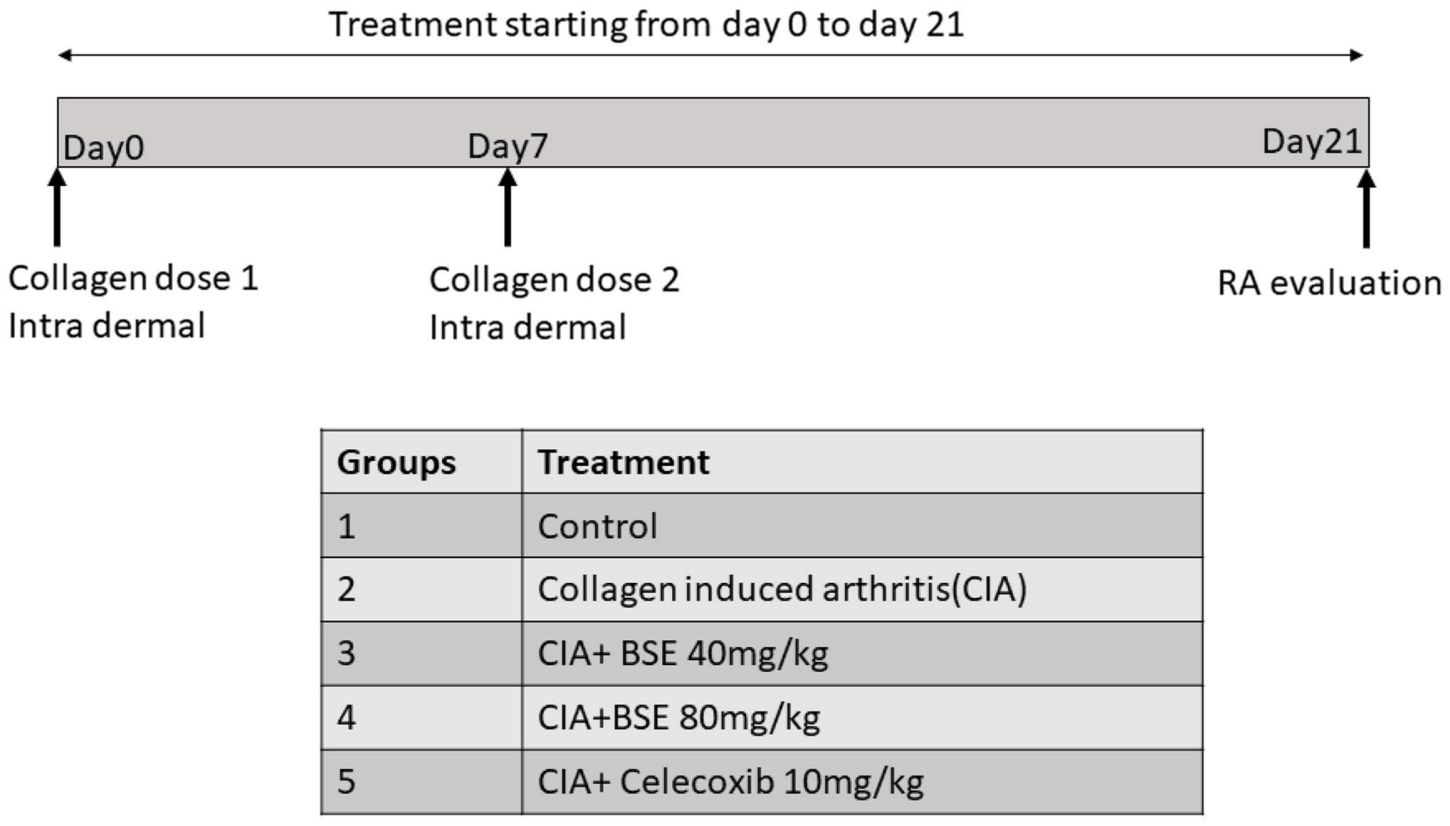


### Evaluation Parameters

#### Arthritic Index Score

A standardized method of arthritis scoring was used to evaluate the degree of swelling and erythema of all four paws. No sign as 0; – Redness without edema as 1, Redness with mild edema as 2, Redness with severe edema as 3, and Redness, severe edema, and stiffness in movement (Yu et al., 2008).

#### Motility Test Score

The motility of the rats was observed for 5min and scored as 0 for walking easily, 1 when the rats walked with little difficulty, toe touching the floor, and 2, when the rats walked with difficulty, avoiding touching the toes of the inflamed paw to the floor (Amdekar et al., 2012).

#### Stair-Climbing Ability

Overnight fasted rats were trained to climb a staircase with steps at 5, 10, and 15cm for 1 week, having water at the second and food at the third step. The climbing ability of the rats was scored as 0 if the rats did not climb. A score of 1 when the rats climbed to the first step, 2, when they climbed into steps 1 and 2, and 3, if the rats climbed all the three steps (Amdekar et al., 2012).

#### Dorsiflexion Pain

The left hind paw of the rats was gently flexed five times at 5s intervals, resulting in a squeak or withdrawal of the leg. The pain was scored as 0 – when rat showed no squeaking and no withdrawal of leg was scored as 0, either squeaking or withdrawal of leg as 1, and both squeaking and withdrawal of leg.

#### Edema Measurement

Digital vernier calipers measured the edema in the paw and ankle joints.

#### Histopathological Assessments

For histological analysis, rats’ hind paws and knee joints were removed and fixed in 4% paraformaldehyde for 24h and decalcified in phosphate-buffered saline containing 10% EDTA. Joints were processed and embedded in paraffin, and 4μm thick sections were taken. The sections were deparaffinized using xylene and prepared for staining by rehydration in a graded concentration of ethanol in water and finally in water (Zhuqian et al., 2019). The slides containing sections were stained by hematoxylin and Eosin for general histology and using Picrosirius red (0.1% Sirius red in saturated picric acid solution) for 60min, followed by washing with 0.5% acetic acid to visualize collagen (Schmitz et al., 2010). Sections were examined at 10x and 40x magnification in a research microscope using brightfield mode (Nikon Eclipse). The collagen area fraction was calculated by dividing the collagen area by the total tissue area.

#### Estimation of Hyaluronan in Synovial Fluid

Hyaluronan was estimated using a commercial kit (Quantikine Hyaluronan Immunoassay Kit, R&D Systems). The synovial fluid was diluted 300 times to get the hyaluronan concentration within the standard curve. The concentration of hyaluronan was calculated based on the standard provided in the kit. The results were expressed as hyaluronan concentration/mg protein of synovial fluid.

#### ESR and CRP

ESR was measured using ESR analyzer (HumaSRate 24PT. Medsource, Ozone Biomedicals, United States), and CRP was estimated using a commercial kit (Sigma-Aldrich, United States).

#### Serum Collagen Antibody Estimation

Collagen antibody estimation was done by following the standard estimation methods. The diluted serum samples were incubated in collagen-coated plates for 90min at 37°C; the plates were washed with PBST (0.05% tween) and then subjected to differential washing. Anti-CII antibodies were detected using horseradish peroxidase-conjugated anti-rat-IgG (Sigma Chemical Co., St. Louis, Mo.) diluted 1:1000 and incubated for 90min at 37° C. The reaction was revealed with a substrate solution consisting of TMB substrate. The reaction was stopped after incubation for 20min at room temperature with 2N H_2_SO_4_ and absorbance read at 450nm (Yasmin et al., 2014).

#### Estimation of Comp Levels in Synovial Fluid

COMP was estimated using a commercial kit (ELISA kit, NOVUS Biologicals, Colorado, United States). The synovial fluid was diluted three times to get the COMP concentration within the standard curve. The concentration of COMP was calculated based on the standard provided in the kit. The results were expressed as pg/mg protein of synovial fluid.

### Statistical Analysis

All the data are presented as mean±SD. The *in vitro* experiments were repeated thrice in duplicates while animal experiments were carried out with N=6 animals per group. The data were analyzed using the one-way ANOVA followed by the Turkey multiple comparisons test. A value of *p*<0.05 was considered statistically significant in comparison with disease control.

## Results

### *B. serrata* Extract

**Figure 1:**
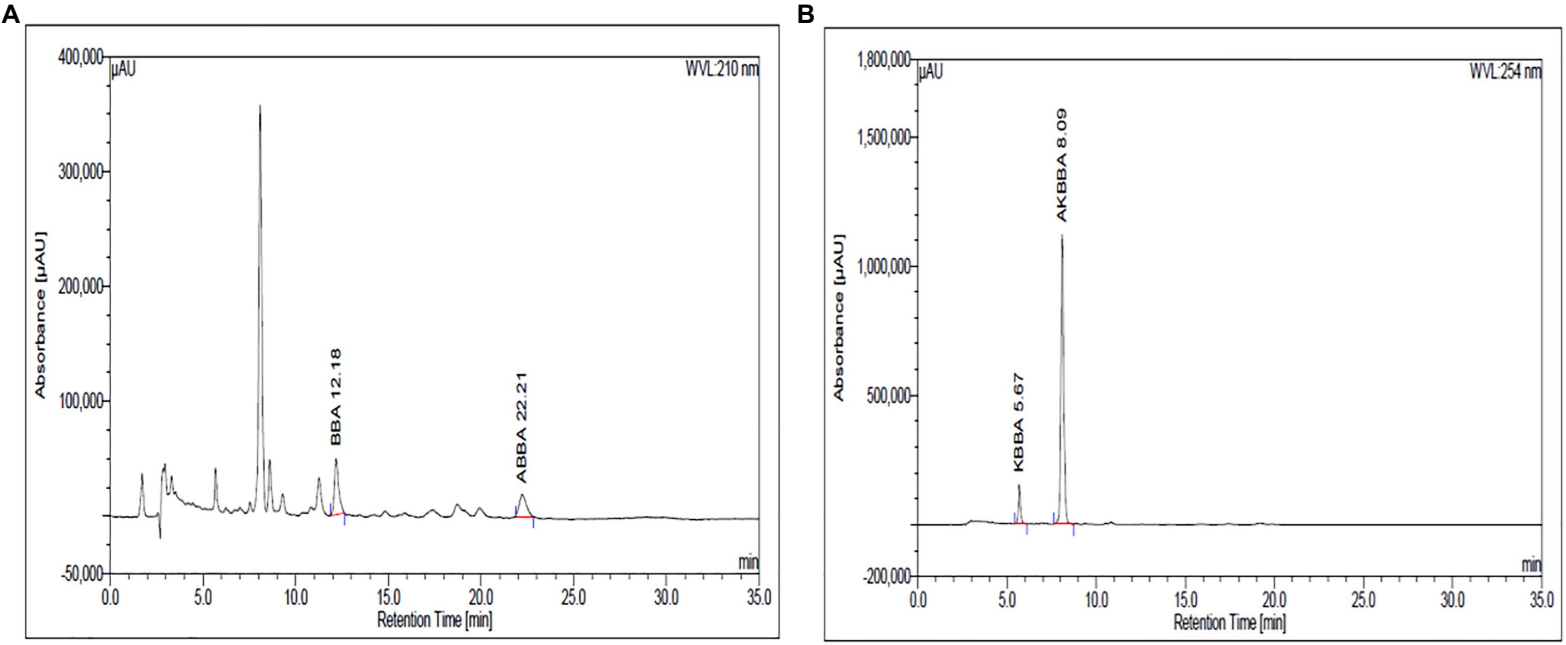
The β-Boswellic acids in BSE were analyzed by HPLC using a C18 column and a mobile phase of acetonitrile and water in the ratio of 90:10 with 0.05ml of glacial acetic acid. BBA and ABBA are detected at 210nm **(A)** and KBBA and AKBBA at 254nm **(B)**.

Boswellin^®^ Super (BSE) is a standardized extract of *B. serrata.* BSE contains at least 30% 3-O-Acetyl-11-Keto- beta-boswellic acid, 7.5% of beta-boswellic acid, 3.5% of 3-O-Acetyl-beta-boswellic acid (ABBA), and 1.5% 11-Keto-beta-boswellic acid (KBBA) as analyzed by HPLC. The content of the total identified beta-boswellic acids was between 50 and 55% in the extract. The HPLC chromatograms are shown in Figures 1A,B, wherein BBA and ABBA are detected at 210nm and KBBA and AKBBA at 254nm.

### Effect of BSE on Antioxidant and Anti-inflammatory Markers *in vitro*

**Figure 2:**
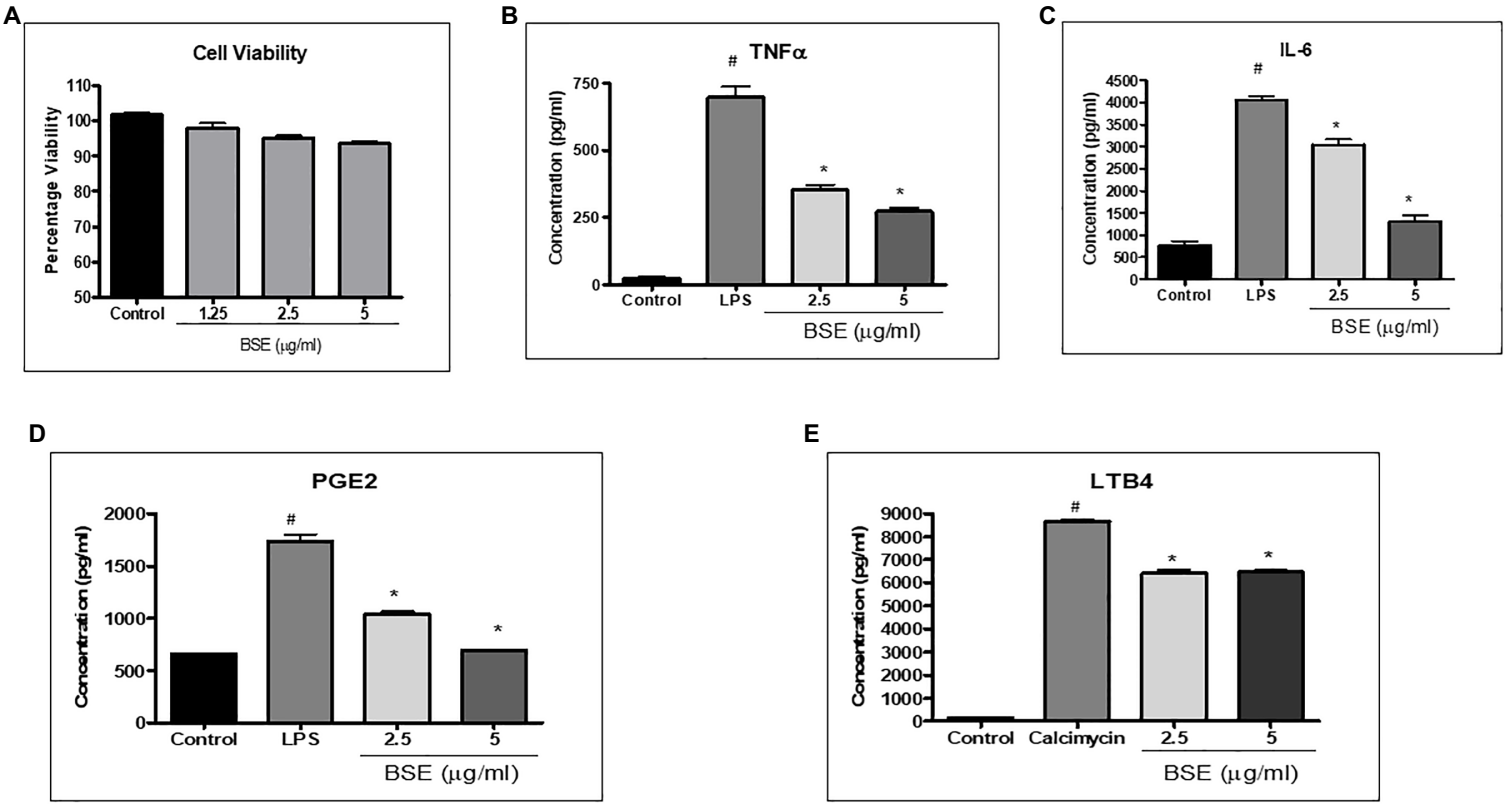
**(A)** Viability of PMBC cells in the presence of different concentrations of BSE effect of BSE on **(B)** TNF-α, **(C)** IL-6, and **(D)** PGE2 in the supernatant of peripheral blood mononuclear cells (PBMC) activated with bacterial lipopolysaccharides, and **(E)** LTB4 in the supernatant of peripheral blood mononuclear cells (PBMC) induced with Calimycin. ^#^ *p*<0.05 compared to control; ^*^ *p*<0.05 compared to the negative control (LPS for TNF-α, IL-6, and PGE2 and Calimycin for LTB4).

**Figure 3:**
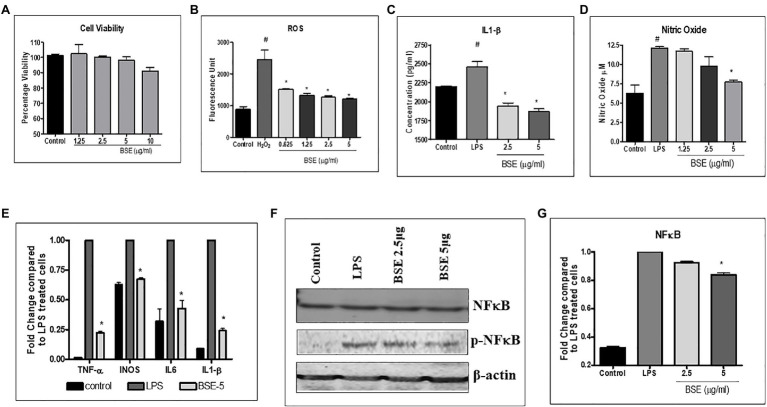
**(A)** Viability of RAW macrophage cells in the presence of different concentrations of BSE **(B)** effect of BSE on intracellular reactive oxygen species (ROS) in RAW 264.7 macrophage cells induced with 25mM hydrogen peroxide (H_2_O_2_). **(C)** IL-1β and **(D)** nitric oxide (NO) in the supernatant of RAW 264.7 macrophage cells induced with LPS (500ng/ml). **(E)** Effect of BSE on relative gene expression of inflammatory biomarkers iNOS, TNF-α, IL-6, and IL-1β in LPS (50ng/ml) induced RAW 264.7 macrophage cells. **(F)** and **(G)** effect of BSE on relative protein expression of inflammatory biomarker p-NFκB in LPS (50ng/ml) induced RAW 264.7 macrophage cells. ^#^ *p*<0.05 compared to control; ^*^ *p*<0.05 compared to negative control H_2_O_2_ for ROS and LPS for IL-1β and NO.

The cell viability of PBMC and RAW 264.7 was not affected at concentrations up to 5μg/ml (Figures 2A, 3A); hence, all the cell-based assays were carried out at a maximum concentration of 5μg/ml of BSE. The secretion of inflammatory cytokines TNF-α and IL-6 from LPS stimulated human PBMC was inhibited in a dose-dependent manner by BSE treatment. At the 5μg/ml concentration, BSE showed 61.1% inhibition of TNF-α and 67.6% inhibition of IL-6 induced by LPS (Figures 2B,C). Significant inhibition was observed in the production of PGE2 (40%) induced by LPS and LTB4 (25.9%) induced by Calimycin in PBMC (Figures 2D,E).

As shown in Figure 3B, treatment with BSE caused a dose-related inhibition in the production of intracellular ROS induced by H_2_O_2_ in RAW 264.7 macrophage cells. The levels of IL-1β (26.5%) and nitric oxide production (38%) were reduced by BSE treatment in RAW 264.7 macrophages stimulated with LPS (Figures 3C,D). Concurrent with these results, the mRNA levels of iNOS TNF-α IL-6 and IL1-β were downregulated in LPS stimulated cells treated with 5μg/ml BSE (Figure 3E). We did not observe any change in IL-8 expression in BSE-treated cells. The protein levels of phosphorylated-NF-κBp65 were also lowered by BSE in the same cells (Figures 3F,G), suggesting an anti-inflammatory activity *via* inhibiting the p-NF-κB signaling cascade.

### Effect of BSE on Extracellular Matrix Protein and Degrading Proteases

The proteins, such as collagen, hyaluronan, and elastin present in the extracellular matrix, provide lubrication, absorb, and distribute the compressive load, and withstand shear stress during joint movement. To understand the effect of BSE on matrix-degrading enzymes, we evaluated the inhibition of collagenase, hyaluronidase, and elastase enzymes by BSE, *in vitro*. Further, its effect on matrix metalloprotease (MMP)-9, the protease that degrades gelatin and collagen, was assessed by zymography.

**Figure 4:**
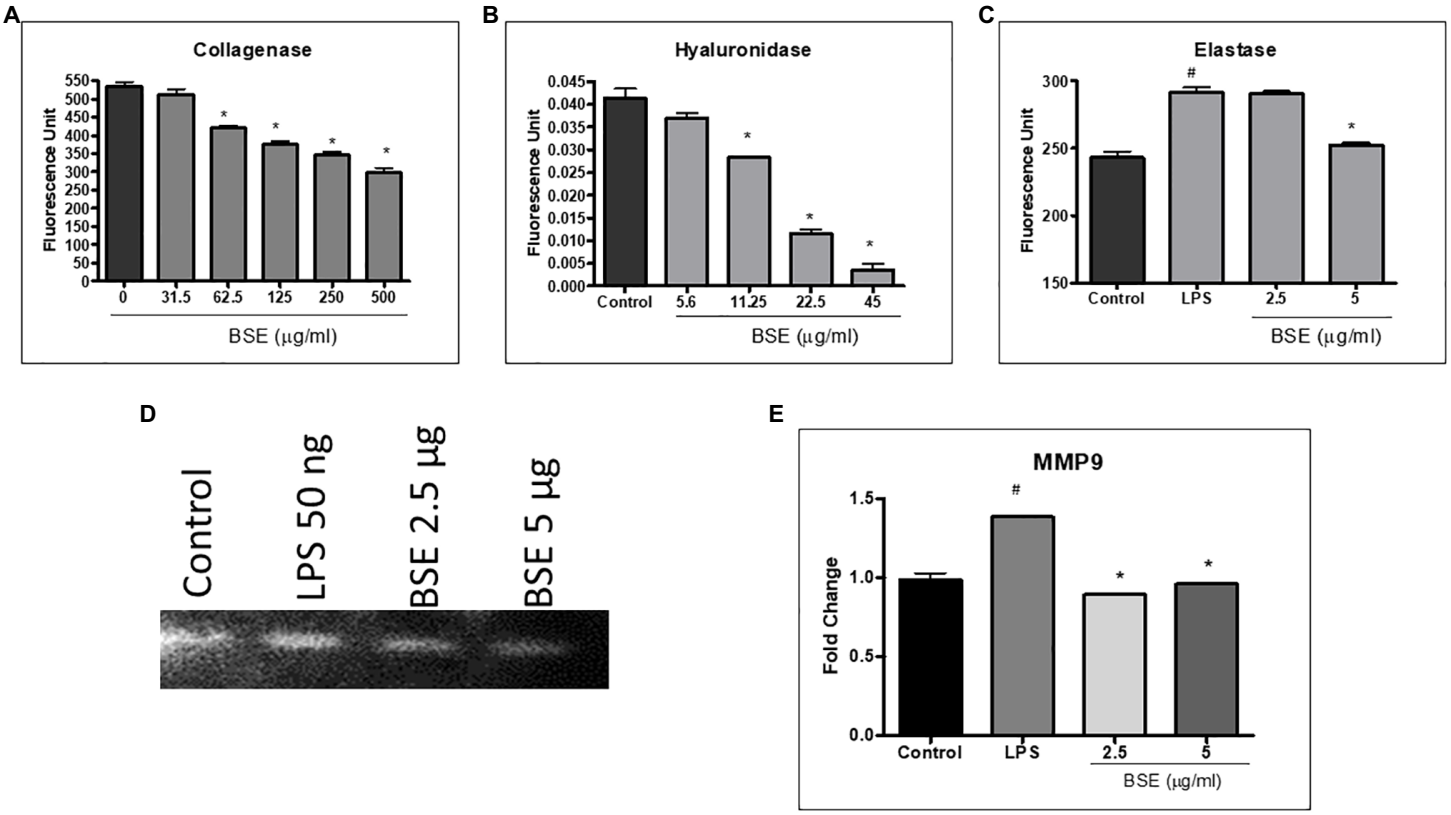
Effect of BSE on **(A)** collagenase enzyme inhibition, **(B)** hyaluronidase enzyme inhibition, and **(C)** elastase enzyme inhibition. **(D)** and **(E)** MMP-9 in cell lysates of RAW 264.7 macrophage cells induced with LPS detected by zymography. ^#^ *p*<0.05 compared to control; ^#^^#^^#^ *p*<0.005 compared to control; ^*^ *p*<0.05 compared to the negative control (vehicle control for collagenase and hyaluronidase enzyme inhibition and LPS for elastase enzyme inhibition and MMP9).

BSE showed dose-dependent inhibition of collagenase enzyme activity with 45% inhibition at a concentration of 500μg/ml (Figure 4A). It showed potent inhibition of hyaluronidase activity, with an IC_50_ of 13.07μg/ml and moderate inhibition of elastase (Figures 4B,C). The enhanced expression of MMP-9 induced by LPS (50ng/ml) was reduced by 5 and 2.5μg/ml of BSE extract (Figures 4D,E).

### Effect of BSE on Collagen-Induced Arthritis in Rats

**Figure 5:**
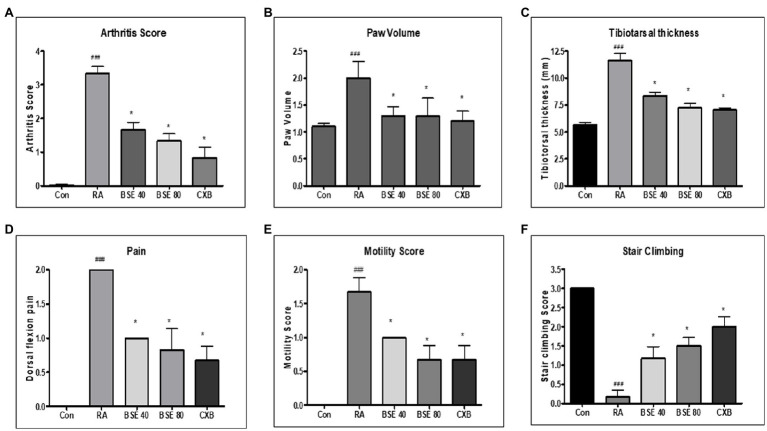
**(A)** Effect of BSE on arthritis index, **(B)** paw volume in collagen-induced arthritis. **(C)** tibiotarsal thickness, **(D)** dorsiflexion pain, **(E)** motility score, and **(F)** stair climbing in collagen-induced arthritis in rats. A standardized method of arthritis scoring was used to evaluate the degree of swelling and erythema of all four paws. The motility pattern, stair climbing, and pain were scored as described in the methods section. ^#^^#^^#^ *p*<0.005 compared to control; ^*^ *p*<0.05 compared to RA animal, BSE 40 and BSE 80, Boswellin Super Extract 40 and 80mg/kg; CXB, celecoxib 10mg/kg; and RA, CIA control.

The collagen immunized animals showed a significant increase in the arthritic index compared to control animals. The arthritic index showed a significant decrease in animals treated with BSE (49.9 and 59.9% at 40 and 80mg/kg) and celecoxib (74.8%; Figure 5A). The arthritic rats showed a significant increase in paw inflammation, measured in terms of increased volume. BSE reduced the paw volume by 36.6 and 37.5% at 40 and 80mg/kg, which was comparable to celecoxib (40%, *p*<0.05) for both treatments (Figure 5B). The joint thickness almost doubled in RA animals compared to control animals, which was significantly reduced by 28.2 and 37.8% (*p*<0.05) at 40 and 80mg/kg by BSE in comparison with the positive control drug, celecoxib (Figure 5C). The dorsiflexion pain test showed that all the animals in the RA group had severe pain and were unable to bear the flexing of the paw. Treatment with BSE significantly reduced pain by 50.0 and 58.3% (*p*<0.05) at 40 and 80mg/kg (Figure 5D), whereas celecoxib showed a 66.5% reduction.

Due to inflamed paws, the motility of the arthritic animals was severely impaired, and the untreated RA-induced animals walked with difficulty. BSE eased motility by 40.1 and 59.8% (*p*<0.05) at 40 and 80mg/kg. BSE at 80mg/kg was equivalent to celecoxib (59.8%) treatment (Figure 5E).

In the stair-climbing test, animals with RA were unable to climb stairs due to inflamed paws, while the control animals easily climbed all three steps. BSE improved the climbing ability by 38.9 and 50.0% at 40 and 80mg/kg, compared to celecoxib (66.67%; Figure 5F).

### Histological Evaluation

**Figure 6:**
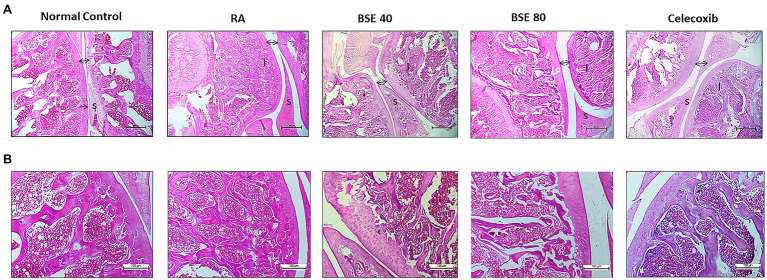
**(A)** H- and E-stained histopathological images of a typical ankle joint of each group. A, 4X and **(B)** 10X resolution. S, synoviocytes and synovial hyperplasia in diseased control. A double arrow indicates space between cartilage bone of joints, and increased space between bone cartilage was found in diseased control; “I” indicates inflammation in the bone marrow. BSE 40 and BSE 80, Boswellin Super Extract 40 and 80mg/kg; CXB, celecoxib 10mg/kg; and RA, rheumatoid arthritis.

Histological examination of the ankle joints of rats from different groups further supported the therapeutic effect of BSE on CIA. As shown in Figure 6, the ankle joint of the control group showed intact bone architecture and articular cartilage, open joint space, and absence of inflammatory cell infiltration. The RA model showed pathological changes with bone erosion, inflammatory cell infiltration, and narrow joint space. BSE and celecoxib treatments restored the morphology of bone and cartilage structure. Further, an alleviation of inflammatory cell infiltration and synovial hyperplasia was observed in the treated animals. BSE downregulated the total pathological score, which was comparable to celecoxib (Table 1).

**Table 1:** Histopathological scores of ankle issues.

Group	Synovial tissue hyperplasia	Inflammatory cell infiltration	Articular cartilage erosion
Control	0	0	0
RA	9.33±0.33	9.17±0.31	9.17±0.31
Celecoxib 10	6.17±0.48^*^	4.83±0.31^*^	5±0.37^*^
BSE40	6.0±0.26^*^	6.67±0.33^*^	7.5±0.43
BSE80	1.50±0.22^*^	5±0.37^*^	5.67±0.56^*^

BSE40 and BSE80, Boswellin^®^ Super 40 and 80mg/kg; CXB, celecoxib 10mg/kg; and RA, rheumatoid arthritis. ^*^ *p*<0.05 compared to RA animal.

### Effect of BSE on Preserving Collagen in the Joints

**Figure 7:**
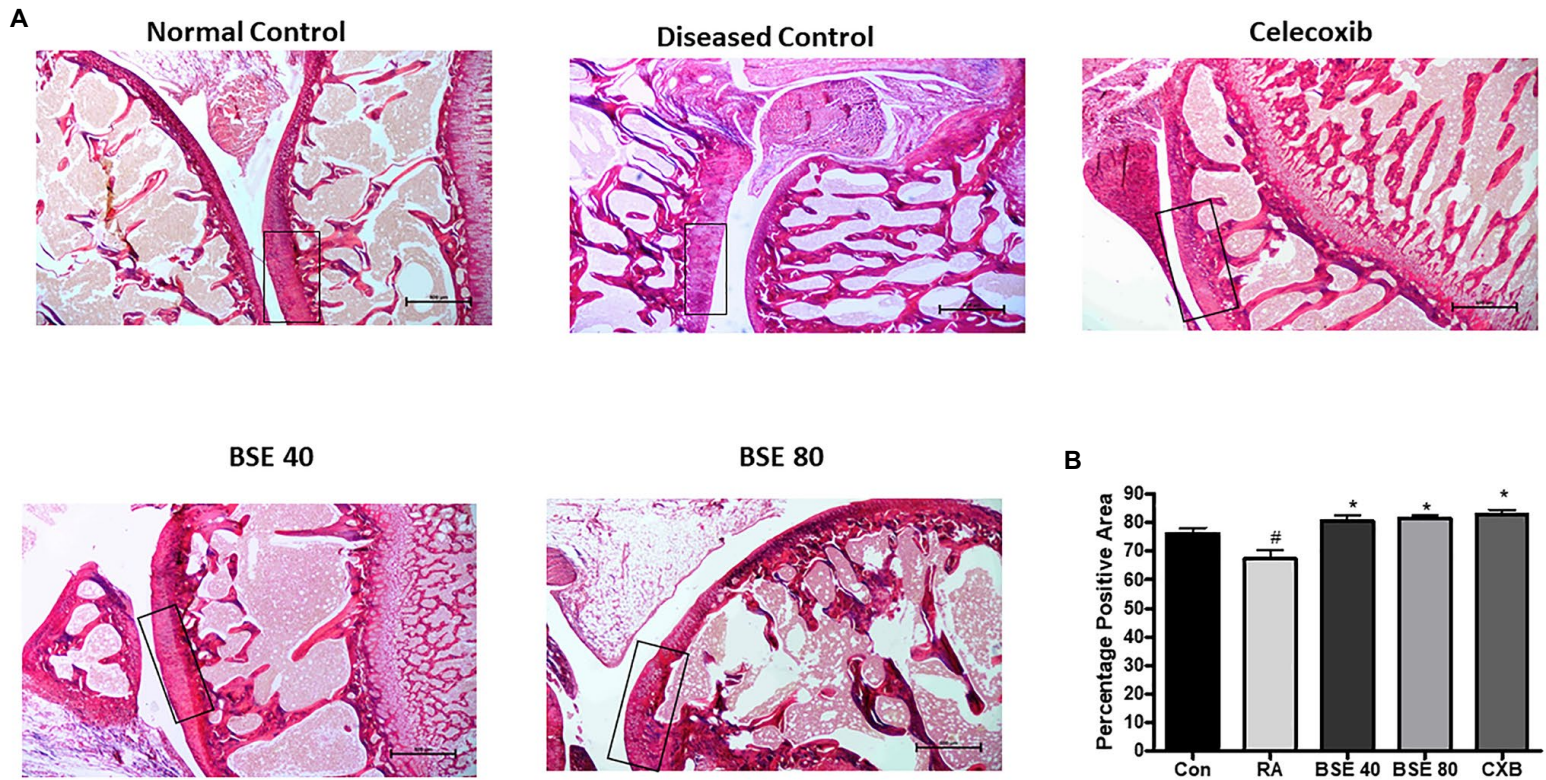
**(A)** Picrosirius stained images of ankle joint. **(B)** Percentage positive area out of total tissue area. The box represents the intact collagen, which is degraded in the CIA control. BSE 40 and BSE 80, Boswellin. Super Extract 40 and 80mg/kg; CXB, celecoxib 10mg/kg; and RA, CIA control. ^*^ *p*<0.05 compared to RA animal; ^#^ *p*<0.005 compared to control.

**Figure 8:**
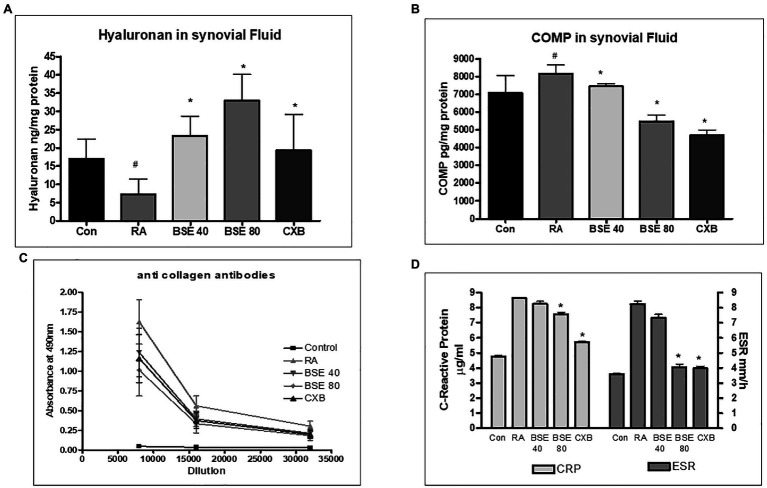
Effect of BSE on **(A)** hyaluronan in synovial fluid and **(B)** cartilage oligomeric matrix protein (COMP) in synovial fluid, **(C)** collagen antibodies in serum, and **(D)** serum C-reactive protein (CRP) and erythrocyte sedimentation rate (ESR) levels in collagen-induced arthritis in rats. ^#^ *p*<0.05 compared to control; ^*^ *p*<0.05 compared to RA animal; BSE40 and BSE80, Boswellin^®^ Super 40 and 80mg/kg; CXB, celecoxib 10mg/kg; and RA, rheumatoid arthritis.

The extracellular matrix of cartilage is composed primarily of type 2 collagen, proteoglycans containing hyaluronic acid, chondroitin sulfate (CS), fibers, fibronectin, and laminin. We evaluated the presence of total collagen in the joints by staining with picrosirius red using a light microscope. Picrosirius red staining is widely used to visualize the distribution of collagen in tissue sections by brightfield or polarization microscopy (Vogel et al., 2015). The loss of collagen in BSE-treated rats was significantly lower compared to CIA control rats, as observed by a higher intensity of picrosirius staining (Figures 7A,B). These results could be correlated with the results of cartilage and bone erosion observed in the H&E-stained sections. Hyaluronan was significantly reduced in arthritic animals, which was restored in BSE-treated rats. The significance was not achieved probably due to variability between the concentrations in the animals (Figure 8A).

The cartilage oligomeric matrix protein (COMP) concentration increased in the synovial fluid of untreated arthritic animals. COMP levels significantly decreased in animals treated with BSE at 80mg/kg (Figure 8B).

### Effect of BSE on Inflammatory and Matrix-Degrading Markers in Rats

Collagen-induced arthritis elicits anti-collagen antibodies in rats. ELISA was used to evaluate the presence of anti-collagen antibodies in serum. Animals with RA had very high levels of antibodies to collagen. BSE treatment reduced the collagen antibodies in the serum. The effect of BSE at 80mg/kg was better than celecoxib (Figure 8C).

C-reactive protein concentration in serum and erythrocyte sedimentation ratio increased in the untreated arthritic animals, which were reduced in animals treated with BSE (Figure 8D).

## Discussion

The present study demonstrates that BSE, a standardized extract of *Boswellia serrata* containing 30% 3-acetyl-11-keto-β-boswellic acid, reduces inflammation, oxidative stress and, most importantly, preserves the matrix proteins by inhibiting the enzymes which hydrolyze these proteins.

BSE showed a reduction in clinical signs of joint swelling, which could be correlated with its anti-inflammatory activity. RA is an inflammatory disease characterized by chronic and persistent inflammation of synovial membranes, causing cartilage destruction and bone erosion (Zhao et al., 2015). Although recent medications have largely improved the course of the disease, they are associated with notable side effects. Hence alternative and complementary medicine is sought to relieve the symptoms and reduce the side effects of drugs. Extracts from *Boswellia serrata* gum resin, with different compositions of boswellic acids, have been used to treat a variety of inflammatory conditions like arthritis and inflammatory bowel disease (Ammon, 2016). While joint pain is a common symptom of OA and RA, the etiology of the two diseases is highly different. OA is a degenerative disease, while RA is an immune disease with the involvement of a self-destructive inflammatory immune response.

Inflammatory mediators, such as TNF-α, IL-6, and PGE-2, play a pivotal role in synovial hyperplasia observed in RA (Takayanagi, 2007). Downregulation of these inflammatory components, consequently, reduces the severity and progression of the disease. The stimulation of the production of TNF*-α* and NO could be linked to the activation of the NF-*κ*B. Suppressing the NF-κB transcriptional activity in the macrophages is beneficial to suppress the expression of iNOS, COX-2, 5-LOX, and other inflammatory pathways (Peng et al., 2011). BSE markedly inhibited the phosphorylated-NF-*κ*B (p65), suggesting that the anti-inflammatory activity is mediated by suppressing the key signal-transducing protein. NO is synthesized in excess from the synovium of inflamed joints and exacerbates joint damage and causes T-cell dysfunction contributing to bone loss in patients with RA (Lever, 2001). BSE significantly inhibited NO levels in cell supernatant and gene expression of iNOS in a dose-dependent manner. Apart from the anti-inflammatory activity, BSE could also quench the intracellular ROS in macrophages. Oxidative stress and ROS can activate various signaling pathways having a vital importance in the pathophysiology of RA (Phull et al., 2018). Excessive ROS can cause direct or indirect damage to the cartilage, matrix proteins, and DNA.

Collagen-induced arthritis, which shares the immunological and pathological features of human RA, is the most widely used animal model for the evaluation of novel therapeutic strategies for RA. BSE showed a reduction in clinical features of RA in the mice model comparable to the effect of the nonsteroidal inflammatory drug celecoxib. The main pathological characteristics of RA are inflammatory cell infiltration and pannus formation, causing joint inflammation, deformity, and the loss of motility and activity. BSE treatment reduced systemic inflammation and the swelling in joints essentially by reducing inflammation. The efficacy of BSE at 80mg/kg was comparable to celecoxib for most of the parameters studied.

The immune system is involved in the progression and maintenance of inflammation in RA. Collagen antibodies reacting to type II collagen in the cartilage were observed to be associated with disease severity, higher levels of inflammatory cytokines in serum and erythrocyte sedimentation ratio (Cook et al., 1996; Bevaart et al., 2010). BSE could reduce the serum levels of anti-collagen antibodies, which could be correlated with the significant reduction in inflammation and arthritis symptoms.

Cartilage degradation is an important pathology of RA. The synovial membrane can be divided into two layers, the continuous surface layer of cells (intima), consisting of fibroblast-like synoviocytes and macrophages, and the underlying subintimal issue having resident fibroblasts and infiltrating cells in a collagenous extracellular matrix (Smith, 2011; Smith and Wechalekar, 2015). The synovial cells secrete hyaluronic acid for lubrication in a normal joint and maintain homeostasis in matrix synthesis and degrading enzymes. Hyperplasia of intimal cells results in increased production of matrix metalloproteinases (MMP) which degrade the ECM of cartilage and synovial membrane (Siebuhr et al., 2012). The inflammatory cytokines from hyperplasic synovium also perpetuate joint damage (Guo et al., 2018). The MMP mediated degradation of extracellular matrix proteins release COMP, and therefore, it is suggested to be a marker of disease progression with prognostic value (Kullich et al., 2006). Reduction of COMP in BSE-treated rats could be beneficial in minimizing joint destruction, which will be highly beneficial for RA patients. Apart from these markers, hyaluronan plays a significant role in protecting the articular cartilage by blocking the loss of proteoglycans and helps in nutrient transport. In RA, hyaluronan additionally acts as an anti-inflammatory molecule by inhibiting the adherence of immune complexes to neutrophils and protects the synovial tissues from the attachment of inflammatory mediators (Tamer, 2013). Enhanced hyaluronan levels in synovial fluid further support the role of BSE in protecting the cartilage in RA rats. Interestingly, the increase in hyaluronan levels by BSE was superior in celecoxib, although it was not statistically significant. In substantiation with these results, BSE inhibited the activity of hyaluronidase and MMP9 enzyme activity *in vitro*, which could be the mechanism of action of BSE in increasing hyaluronan in the synovial fluid. Further, BSE treatment significantly inhibited collagenase and elastase enzyme activities *in vitro*, suggesting that BSE could reduce the ECM degradation and preserve the extracellular matrix. Further, the total collagen content was higher in BSE-treated animals, suggesting ECM integrity.

The histopathological observations of the joints further supported the results observed in the alterations in the biomarkers. Increased infiltration of inflammatory cells, the congestion and hyperplasia of synovium, the damage of cartilage, and bone erosion were observed in the CIA rats, while BSE treatment significantly reduced these changes.

In summary, these results suggest that the anti-arthritic effect of BSE is mediated by regulating pro-inflammatory cytokines, nitric oxide, and oxidative stress along with preserving matrix proteins and reducing joint damage, which are the key markers for RA. We have earlier demonstrated the efficacy of BSE (standardized extract with 30% AKBBA) in mitigating clinical symptoms in osteoarthritic patients (Majeed et al., 2019). These results suggest that BSE could be a potential supplement to reduce the pain and inflammation associated with rheumatoid arthritis. In RA, recurrence of symptoms (flares) while tapering DMARDs is a common occurrence. The clinical trial with BSE as an adjunct therapy would initially be aimed at prolonging remission and stabilizing the disease. Larger trials can be planned based on the preliminary results of this trial.

## Conclusion

Based on the results, it can be concluded, BSE has a significant effect on RA in a collagen-induced autoimmune arthritis model. The anti-arthritic effect was comparable to the standard anti-inflammatory drug celecoxib at higher concentrations. Thus, BSE may be considered as a promising supplement for managing RA in a clinical setting. Future clinical trials are warranted to translate the preclinical results to human use.

## Data Availability Statement

The original contributions presented in the study are included in the article/supplementary material, and further inquiries can be directed to the corresponding author.

## Ethics Statement

The animal study was reviewed and approved by the Committee for the Purpose of Control and Supervision of Experiments on Animals (CPCSEA), India, with the approval from the Institutional Animal Ethics Committee (approval no. 1165/PO/RcBiBt-S/NRc-L/08/CPCSEA).

## Author Contributions

MM and KN: conceptualization. LM and KN: methodology. LL, RN, and VT: formal analysis, investigation, and writing—original draft preparation. MM: resources. LM and RN: data curation. LM, KN, and MM: writing—review and editing. LM: supervision. All authors have read and agreed to the published version of the manuscript.

## Conflict of Interest

All authors are employees of the Sami-Sabinsa Group Limited and Sabinsa Corporation. Boswellin^®^ Super is marketed by the Sami-Sabinsa Group Limited and Sabinsa Corporation.

## Publisher’s Note

All claims expressed in this article are solely those of the authors and do not necessarily represent those of their affiliated organizations, or those of the publisher, the editors and the reviewers. Any product that may be evaluated in this article, or claim that may be made by its manufacturer, is not guaranteed or endorsed by the publisher.
